# Exosome-associated AAV2 vector mediates robust gene delivery into the murine retina upon intravitreal injection

**DOI:** 10.1038/srep45329

**Published:** 2017-03-31

**Authors:** Sarah J. Wassmer, Livia S. Carvalho, Bence György, Luk H. Vandenberghe, Casey A. Maguire

**Affiliations:** 1 Harvard Stem Cell Institute, Harvard University, Cambridge, MA, USA; 2 Grousbeck Gene Therapy Center, Schepens Eye Research Institute and Massachusetts Eye and Ear, Boston, MA, USA; 3 Ocular Genomics Institute, Department of Ophthalmology, Harvard Medical School, Boston, MA, USA; 4 Department of Neurobiology and Howard Hughes Medical Institute, Harvard Medical School, 220 Longwood Avenue, Boston, 02115 MA, USA; 5 Department of Neurology, Massachusetts General Hospital and NeuroDiscovery Center, Harvard Medical School, Building 149, Charlestown, Boston, 02129 MA, USA

## Abstract

Widespread gene transfer to the retina is challenging as it requires vector systems to overcome physical and biochemical barriers to enter and diffuse throughout retinal tissue. We investigated whether exosome-associated adeno-associated virus, (exo-AAV) enabled broad retinal targeting following intravitreal (IVT) injection, as exosomes have been shown to traverse biological barriers and mediate widespread distribution upon systemic injection. We packaged an AAV genome encoding green fluorescent protein (GFP) into conventional AAV2 and exo-AAV2 vectors. Vectors were IVT injected into the eyes of adult mice. GFP expression was noninvasively monitored by fundus imaging and retinal expression was analyzed 4 weeks post-injection by qRT-PCR and histology. Exo-AAV2 outperformed conventional AAV2 in GFP expression based on fundus image analysis and qRT-PCR. Exo-AAV2 demonstrated deeper penetration in the retina, efficiently reaching the inner nuclear and outer plexiform, and to a lesser extent the outer nuclear layer. Cell targets were ganglion cells, bipolar cells, Müller cells, and photoreceptors. Exo-AAV2 serves as a robust gene delivery tool for murine retina, and the simplicity of production and isolation should make it widely applicable to basic research of the eye.

Due to their high efficiency of gene transfer *in vivo* and an overall favorable safety profile, AAV has become a preferred therapeutic gene delivery vector, now reaching validation in several clinical trials. Retinal gene therapy programs have led the field, due the compartmentalized nature of the eye, its relative immune privilege, and low dose requirement^1^,^2^,^3^. The two major injection routes to deliver transgenes to the retina are subretinal (SR) and intravitreal (IVT). An SR injection delivers a suspension between the photoreceptor layer and the retinal pigment epithelium (RPE). In doing so, the retina is detached from the back of the eye, however animal and human experience demonstrates this to resolve in a matter of days^4^,^5^. Vector delivery via SR henceforth allows diffusion and transduction of the RPE and photoreceptor layers, however only in a focal area around the site of injection^6^. Generally well tolerated, the impact of the detachment on long term safety remains debated. The murine eye is small with a diameter of 3 mm and retinal area of 16 mm^2^ in comparison to a human eye of 28 mm diameter and 1000 mm^2^ retinal surface. Consequently, SR injections are difficult to perform precisely and reproducibly in mice. On the contrary, an intravitreal injection (IVT) is less invasive and since an agent is injected directly into the vitreous humour it may permit more broad and uniform retinal targeting^7^. AAV transduction following IVT however is typically restricted to outer retinal cell layers, predominantly retinal ganglion cell (RGC), the cell type most proximal to the site of injection^8^. Moreover, even at high doses, transduction is limited due to a number of barriers for transduction that remain to be fully defined; a physical barrier is created by the vitreous humour^9^, the inner limiting membrane (ILM)^10^, and the complex tangle of different cells and processes that form the inner retina which the vector has to be able to circumvent to reach the photoreceptors in the outer nuclear layer (ONL). Efforts towards mitigation of these barriers has been investigated and shown that mild enzymatic digestion of the ILM with Pronase does improve transduction of the multiple cell types in the retina, with the most robust expression with AAV5 serotype^11^. Other factors, such as post-cellular entry steps (e.g. proteasome-mediated degradation) are thought to be another barrier to efficient retinal transduction^8^.

Exosomes represent a promising novel drug and gene delivery vehicle^12^. These lipid vesicles are secreted by all types of cells and can transfer proteins and RNA^13^. Recently we have shown that AAV associates with exosomes^14^ and that exosome-associated AAV (exo-AAV) vectors represent a novel gene delivery vector with several advantageous properties^15^,^16^. Exo-AAV vectors outperformed conventional AAV vectors in transduction *in vitro* and *in vivo* and exhibited marked resistance to neutralizing antibodies. Since exosomes can cross the blood brain barrier^17^,^18^, and we have shown exo-AAV to cross an endothelial barrier^16^, we hypothesized that exosomes might also facilitate penetration of AAV vectors across other barriers, such as between the vitreous and the retina.

Therefore, in this study our aim was to investigate the potential of exosome-associated AAV to enhance vector transduction of the retina from the intravitreal route. We demonstrate that exosome-associated AAV2 vectors highly outperform conventional AAV2 in retinal transduction after intravitreal injection and are able to transduce high number of bipolar cells and also some photoreceptors.

## Results

### AAV2 capsids are closely associated with exosomes isolated from 293T AAV-producer media

**Figure 1:**
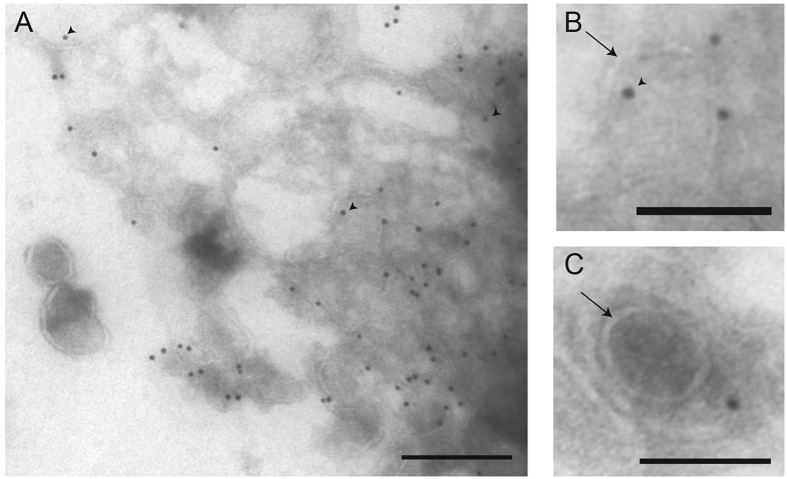
AAV2 associates with exosomes pelleted from AAV-producing 293T cell culture media. Transmission EM images show exo-AAV2 particles with immunolabeling against AAV capsids. Arrows show exosomal membrane. Immunolabeling is present mostly on the outer surface of exosomes, however there is some immunolabeling inside lipid vesicles (arrowheads). Scale bars are 200 nm (**A**) and 100 nm (**B**,**C**).

Before assessing the function of exosome-associated AAV2 (exo-AAV2) for genetic modification of murine retina after IVT delivery, cell culture media from AAV2-producing 293T cells was subjected to ultracentrifugation and the exosome pellet was analyzed with transmission electron microscopy (TEM) using immunogold labeling with an antibody which recognizes intact AAV2 capsids. We observed several lipid vesicles between 50–300 nm in diameter corresponding to exosomes and larger microvesicles. Immunogold labeling on the exosome membrane/interior of some vesicles confirmed the presence of associated AAV capsids (Fig. 1). Immunogold labeling showed mostly surface localization of AAV, however, there was some labeling likely on the interior of exosomes as well (Fig. 1A, arrowheads). Quantitation of several TEM images revealed 57.2% +/− 9.8% (mean +/− SEM) of labeled AAV capsids were in contact with exosomes. From harvested media from AAV2-producing 293T cells, we quantitated AAV genome copies in the 20,000 × g, 100,000 × g pellets as well as the media after these centrifugations (Table 1). We found that only 6.8% of the AAV2 remained in the media after pelleting steps.

### Intravitreal injection of exo-AAV2 outperform conventional AAV2 in gene delivery

**Figure 2:**
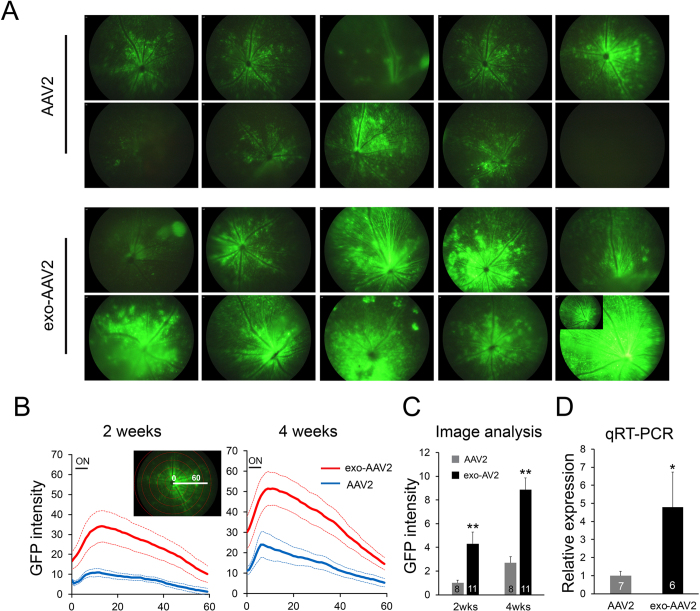
Exo-AAV2 outperforms conventional AAV2 transduction of retina following intravitreal injection (2 × 10^9^ vg/eye). (**A**) Fundus images at 4 weeks post-injection. The inset in one of the eyes injected with exo-AAV2 shows the same fundus image with lower gain. (**B**) GFP intensity quantification of fundus images. Solid lines represent average values at each distance from the optic nerve head, dotted lines represent +/− SEM. ON denotes optic nerve head. Data was gathered from 8 (for AAV2) and 11 (for exo-AAV2) eyes. Numbers on the *x* axis denote arbitrary distance units (arbitrary units, au). Inset shows the image quantification method used (concentric circle plugin in ImageJ). (**C**) Total GFP intensity on the fundus images, **p < 0.01, Mann Whitney U test, numbers in bars represent number of analyzed samples. (**D**) qRT-PCR for GFP mRNA at 4 wks post-injection. Expression was normalized to GAPDH expression level. *p < 0.05, Mann Whitney U test, numbers in bars represent number of analyzed samples.

To compare gene transfer efficiency of exo-AAV2 and conventional AAV2 vectors (referred to simply as AAV2 from this point forward), we injected the vectors encoding self-complimentary (sc) GFP under control of the strong CMV immediate early/chicken beta actin (CBA) hybrid promoter at 2 × 10^9^ vector genomes (vg) into the vitreous of adult C57BL/6 male mice. Table 2 describes these different vector preparations. Transgene expression was evaluated at 2 and 4 weeks post-injection by fundus imaging (Fig. 2A, upper panel). There was inevitable surgical variation between eyes as evidenced by one AAV2 eye that did not show any GFP expression (Fig. 2A, upper panel, last image) and one exo-AAV2 eye with low GFP expression (Fig. 2A, lower panel, first image). All eyes from the study are represented in Fig. 2A. We did not observe any apparent toxic effect of either vector in the anterior and posterior segments of the injected eyes. Using AAV2-scGFP, we detected relatively even retinal GFP expression, which was more prominent around the optic disk and the center of the retina. GFP expression pattern was characteristic to an intravitreal injection with prominent perivascular and granular GFP positivity (Fig. 2A, lower panel). We have also observed GFP expression in the ciliary body using exo-AAV2 (Supplementary Figure S1A–C). While the pattern of expression was similar, intravitreal delivery of exo-AAV2 showed higher GFP expression than AAV2. We quantified GFP expression intensity and found that, both at 2 and 4 weeks, exo-AAV2 led to a higher intensity of GFP expression (Fig. 2B,C). Image analysis confirmed that GFP expression from either vector was the highest around the optic disk, while gradually decreasing towards the periphery. However, exo-AAV2 led to higher GFP expression at all distances from the optic nerve (Fig. 2B). GFP expression intensity increased from 2 weeks to 4 weeks by 50–70%, with exo-AAV2 outperforming AAV2 at both time points. We also quantified total GFP expression on fundus images, and found that exo-AAV2 led to 4.3-fold and 3.3-fold increases in overall GFP expression levels compared to AAV2 at 2 and 4 weeks post-injection, respectively (p = 0.001 for the 2 weeks’ time point, p = 0.009 for the 4 week’s time point, two-tailed Mann Whitney U-test) (Fig. 2C). We went on to further quantify the GFP expression levels by performing qRT-PCR for GFP mRNA in a subset (n = 7 for AAV2, n = 6 for exo-AAV2) of the eyecups (Fig. 2D). GFP mRNA at 4 weeks post-injection was 4.8-times higher in eyes injected with exo-AAV2 than AAV2 and the difference was statistically significant (p = 0.038, two-tailed Mann-Whitney U test). These results demonstrate that exo-AAV2 outperforms AAV2 in retinal transduction after intravitreal injection.

### exo-AAV2 vectors transduces deeper retinal layers compared to AAV2

**Figure 3:**
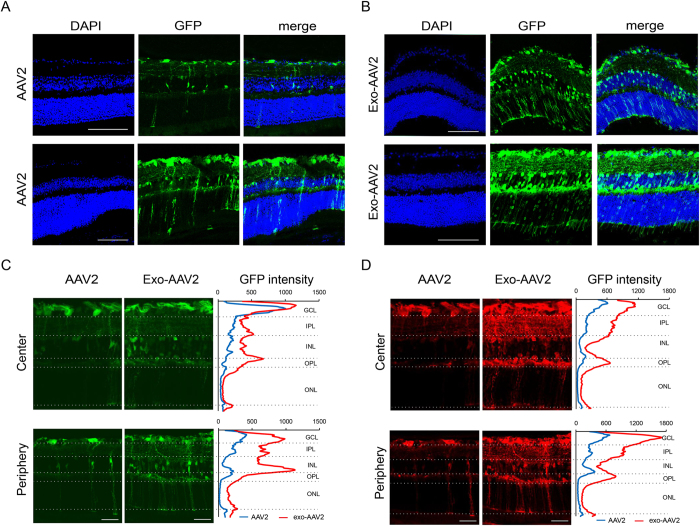
Distribution of transduced cells in the retina after intravitreal injection of exo-AAV2 and conventional AAV2. (**A**,**B**) Two representative sections of retinas from eyes injected with AAV2 (**A**) or exo-AAV2 (**B**), scale bars represent 100 μm. Green channel: direct GFP fluorescence, (**C**,**D**) Quantification of direct GFP fluorescence (**C**) or the signal intensity of GFP immunostaining (**D**) from retinal sections, scale bars represents 40 μm. Images show average intensity *z* projections. Graphs show mean GFP intensity (3 eyes for conventional AAV and 5 eyes for exo-AAV) as a function of distance from the inner limiting membrane. Dotted lines represent the width of different retinal layers. GCL: ganglion cell layer, IPL: inner plexiform layer, INL: inner nuclear layer, OPL: outer plexiform layer, ONL: outer nuclear layer.

To assess the retinal distribution of AAV2 and exo-AAV2 vector mediated viral transduction, we cryosectioned the injected eyes at 4 weeks post-injection for GFP expression analysis. AAV2 transduction was very prominent in retinal ganglion cell (RGC) layer (Fig. 3A), but GFP expression was weaker in deeper retinal layers as reported previously^19^. We could observe some GFP expression in the inner plexiform layer and some scattered cells throughout the outer retinal layers. In contrast, exo-AAV2 showed high GFP expression in several retinal layers, with high GFP intensities in the RGC layer, inner plexiform, inner nuclear and outer plexiform layers (Fig. 3B). There was also evidence for GFP delivery in the outer nuclear layer, but not in the outermost retinal pigment epithelial cell layer. These results suggest that exo-AAV2 vectors can successfully transduce deeper retinal layers compared to AAV2. We quantified the GFP expression intensity on the images by function of the distance from the inner limiting membrane. Figure 3 shows representative images from direct GFP fluorescence (no immunostaining, Fig. 3C) and anti-GFP stained retinas (Fig. 3D). GFP immunostaining revealed more abundant GFP expression compared to what was detectable without staining, particularly in the inner plexiform layer. Exo-AAV2 outperformed AAV2 transduction in most retinal layers, however the difference was the most striking in the deeper retinal layers (Fig. 3C,D). AAV2 transduced mainly retinal ganglion cells and transduction efficiency gradually decreased towards the deeper retinal layers. On the contrary, with exo-AAV2, we observed efficient and similar transduction in RGC as well as in the inner nuclear layer/outer plexiform layer. This analysis confirms that exo-AAV2 is not only capable of enhancing transduction of AAV2 but also of increasing the extent of transduction across different regions of the retina after intravitreal delivery.

### exo-AAV2 transduces a wide cellular profile in the retina

**Figure 4:**
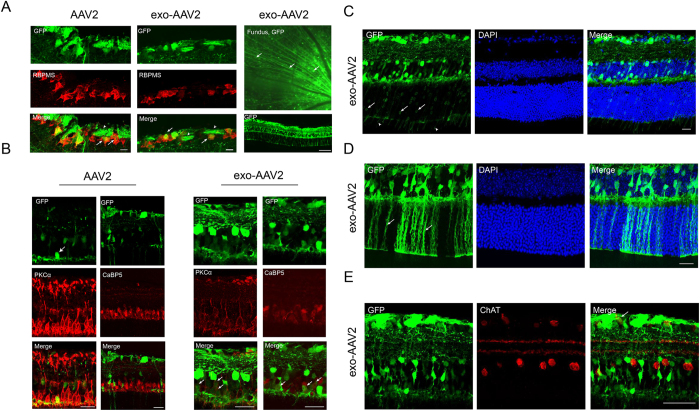
Transduction of different retinal cell types by exo-AAV2 after intravitreal injection. (**A**) Co-localization of GFP and RBMPS staining (retinal ganglion cell marker). Scale bar represents 20 μm. Arrows point to transduced ganglion cells, arrowheads show GFP positive nerve fibers. Right panel shows higher magnification of a fundus image with several nerve fibers positive for GFP (arrows). Lower right section shows a section of the same eye as on the panel above, scale bar represents 100 μm. (**B**) Bipolar cell targeting of exo-AAV2 and conventional AAV2. Bipolar cells were stained with antibodies against PKCα (rod bipolar cells, top) or CaBP5 (all bipolar cells, bottom). Arrows show GFP positive cells, scale bar represents 20 μm. (**C**) Transduction of photoreceptors is detectable by the presence of GFP^+^ cells in the outer nuclear layer (arrows) and inner segments (arrowheads), scale bar represents 20 μm. (**D**) Some areas show prominent Muller cell transduction (arrows), scale bar represents 20 μm. (**E**) ChAT positive amacrine cells did not appear to be GFP positive, scale bar represents 50 μm.

Next we analyzed which cell types are transduced by exo-AAV2 vectors. Not surprisingly, we observed RBPMS^+^ retinal ganglion cells expressing GFP (Fig. 4A, left). The processes of these cells appeared green on the fundus images (Fig. 4A, right) and also in the nerve fiber layer in tissue sections (Fig. 4A, arrowheads). We also analyzed the extent of bipolar cell transduction in the inner nuclear layer. We stained bipolar cells either with PKCα (rod-bipolar marker) or CaBP5 (pan-bipolar cell marker) (Fig. 4B, Supplementary Figure S1D–F). The PKCα stains only a narrow rim of the cell’s cytoplasm, while the CaBP5 stains the entire cell body. Using these two stains, we observed high number of GFP positive bipolar cells in the inner nuclear layer in the exo-AAV2 group. On the contrary, AAV2 transduced only a few bipolar cells (Fig. 4B). We also detected several GFP positive cells in the photoreceptor layer in 4 out of 5 exo-AAV2 injected animals, although the robustness of GFP expression varied between mice (Fig. 4C and Supplementary Figure S2). Photoreceptor transduction was also confirmed by GFP positive inner segments of these cells in the inner segment region (Fig. 4C and Supplementary Figure S2). The animal in which no photoreceptor expression was detected, had an overall lower level of GFP expression throughout the retina, likely indicating a suboptimal injection. We detected several Müller cells (based on morphology) spanning the entire width of the retina (Fig. 4D). Müller cell transduction was confirmed by specific immunostaining (Supplementary Figure S3). In some of the exo-AAV2 samples, it appeared as though there may have been colocalization with ChaT positive amacrine cells, however the staining did not appear highly specific and may have been confounded by the high level of GFP expression throughout the retina (Fig. 4E). We also did not observe any GFP positive ChaT cells in the AAV2 group. We conclude that the majority of cells in the retina have been successfully transduced with IVT administered exo-AAV2 with the exception of the retinal pigment epithelium (RPE) and the ChAT positive amacrine cells.

### In culture transduction properties of exo-AAV2

In order to gain insight to the significant increase in widespread retinal transduction by exo-AAV2 compared to conventional vector, we assayed the dependence of exo-AAV2 on the universal AAV receptor (AAVR)^20^. Using wildtype, AAVR knock-out and AAVR over-expressing cell lines, we performed a luciferase assay to determine the ability for exo-AAV encoding firefly luciferase (FLuc) to transduce AAVR knock-out cells. We found low to background signals from both the exo-AAV2-FLuc and AAV2-FLuc transduced AAVR knock out cells (Supplementary Figure S4). This suggests that exo-AAV2 is also dependent on the universal intracellular AAV receptor for transduction.

We wondered whether the increase in transduction of retinal layers after IVT injection with exo-AAV2 may be due to a general increase in the ability to transduce cells or if it was more specific to particular parameters encountered by the vector *in vivo*. HeLa cells were transduced with an equal dose of either AAV2-FLuc or exo-AAV2-FLuc and transduction analyzed 2 days post incubation (Supplementary Figure S5A). Exo-AAV2 transduction was significantly lower compared to AAV2 transduction (p < 0.001, two tailed t-test), suggesting that the enhancement observed in retinal layer transduction with IVT injected exo-AAV2 may be related to circumventing specific barriers imposed upon AAV2 and not due to a general increase in transduction.

Upon IVT injection, dependence of heparin binding has been implicated in AAV2 being able to cross the ILM and transduce the ganglion cell layer compared to non-heparin binding AAV’s^21^. Our group and others have shown extracellular vesicles to be heparin sensitive in their ability to enter cells^22^,^23^,^24^. We compared the transduction of HeLa cells by AAV2 or exo-AAV2 in the presence or absence of a range of heparin concentrations from 1 to 200 μg/ml. No difference was observed at the doses tested between the two vectors at the heparin concentrations tested (Supplementary Figure S5B).

## Discussion

We investigated retinal transduction of exosome-associated AAV (exo-AAV) vectors injected intravitreally (IVT) into the mouse retina. Conventional AAV vectors only have limited transduction efficiency after IVT injection. In order to reduce surgical invasiveness and potential for surgical complications, and maximize the potential for broad and more homogeneous transduction across the retina, IVT is preferred over the subretinal route of vector administration. However, inefficient transduction with conventional AAVs following IVT injection suggests barriers prevent AAV gene delivery. The vitreous itself may indeed trap vectors due to high viscosity thereby decreasing the effective amount of vector which reaches the retina. This is particularly significant in large animal models; the removal of the vitreous by vitrectomy resulted in enhanced retinal transduction of AAV2 in dogs and macaques^9^. Another important obstacle for transduction appears to be the inner limiting membrane (ILM). Enzymatic digestion of the ILM by non-specific proteases led to robust transduction of the retina by different AAV serotypes in rat^11^. Similarly, retinal transduction was highly enhanced in Dp71 null mice, where the ILM is substantially thinner^25^. Therefore in the clinical setting, surgical methods including vitrectomy or ILM peeling^26^ might be considered to enhance AAV transduction from the vitreous. However, for routine genetic modification of murine retina for basic research, these approaches are not practical.

Another approach to circumvent biological barriers is to engineer or develop novel AAV vectors with certain properties that allow for better transduction from the intravitreal route. In one approach, Dalkara *et al*. performed an *in vivo* with a mixture of different AAV capsid libraries, and identified an enriched capsid clone from a 7-mer peptide AAV2 capsid library (peptides inserted after amino acid 588 in VP1). This capsid clone enhanced transduction of photoreceptors and RPE following IVT injection^27^,^28^. Tyrosine mutant AAV capsids (single, triple or quadruple Y-F) were also shown to result in better retinal transduction after intravitreal injection, likely due to decreased proteosomal degradation of the capsid^29^,^30^,^31^,^32^,^33^. Alternative serotypes such as AAVrh.8 and AAVrh.10 has also shown promise for retinal transduction by the IVT route^34^.

In our study we investigated retinal transduction efficacy of exo-AAV vectors. Several DNA and RNA viruses have been shown to associate with exosomes^35^,^36^,^37^,^38^, possibly through an evolutionally conserved mechanism. Exosome association with AAV was shown previously to confer increased resistance to neutralizing AAV antibodies and possibly influences viral uptake by cells^15^. Exosomes are known to penetrate physical barriers^17^, therefore we hypothesized that exosome association of AAV vectors might also enhance penetration through the ILM. Indeed, our data indicates that exosome association of AAV2 vectors enhances retinal transduction after intravitreal injection, and allows for deeper penetration of the retina. Image analysis on retinal sections revealed that the transduction is not simply enhanced at the ganglion cell layer, but there is also enhanced transduction of deeper retinal layers by exo-AAV2 vectors compared to conventional AAV2 vector. Retinal transduction was highly enhanced with exo-AAV in the INL. When staining for bipolar cells, we observed minimal bipolar cells targeting with conventional AAV2, similarly to previous reports^19^. On the contrary, we achieved robust bipolar cell transduction with relatively modest titers (i.e. 2 × 10^9^ vg/eye) and transduction was evident without staining for GFP. We have also observed photoreceptor cell transduction, particularly at the periphery of the retina. In another study, Petrs-Silva *et al*. found that only high titers of triple or quadruple Y-F AAV vectors (1 × 10^10^ vg/eye) led to targeting of the ONL. Delivering lower amounts of these vectors (1 × 10^9^ vg/eye) only led to RGC and Muller cell transduction with some scattered cells in the inner retina^29^. Therefore, exo-AAV appears to be a promising tool for retinal transduction even at relatively modest doses and it may compete with existing technologies based on comparing our data to that in the literature.

It has been reported that vector injected IVT or subretinally in mice and dogs can transduce regions of brain^39^, thus it will be important to assess whether exo-AAV2 displays the same properties. If we do find brain transduction after IVT injection with exo-AAV2, retina-specific promoters may be used to keep transgene expression restricted primarily to the eye.

The attractiveness of our approach, especially for basic researchers looking to target specific retinal cell types including bipolar cells and photoreceptors via IVT injection can combine cell type specific promoters with exo-AAV2. Similarly, when one seeks to broadly target the retina, exo-AAV2 improve on current gene transfer reagents. The ease of the procedure and the ready availability of reagents adds to the appeal of this technology with (a) AAV2 capsid plasmids without the need for further genetic modification, and (b) in contrast to conventional AAV2 purification which requires highly technical density gradient or affinity chromatography purification, exo-AAV2 is isolated through sequential centrifugations which take less than 2.5 hours to complete. The pelleted AAV and exosomes are resuspended, titered by qPCR, and are ready for injection into murine eye.

Currently, the mechanism of enhanced transduction with exo-AAV or the increased penetration after IVT administration is not understood. Several possibilities exist. For instance, exosomes could enhance the penetration or cellular uptake of AAV vectors in the retina. In general exosomes could be taken up by several parallel mechanisms, including receptor mediated endocytosis, macropinocytosis or phagocytosis^40^ on the contrary to AAV, the uptake of which is restricted to one specific route, depending on the serotype. Therefore exosome association may broaden the uptake of AAV among different cell types. In this study, we found that exo-AAV particles are however, dependent on the universal AAV receptor (AAVR), which is located on the cell surface but highly involved in intracellular trafficking^20^. Therefore it is likely that exo-AAV and AAV use the same intracellular pathways. Other possible mechanisms for increased transduction of retina is increased diffusion out of the vitreous body by exo-AAV. As mentioned, vitrectomy enhances transduction by AAV after IVT injection in dogs and macaques^9^. As observed in our electron microscopy images, there appears to be many AAV-free exosomes which may act to shield the capsid from interactions with vitreous body components. In the future, it may be interesting to test whether vitrectomy has the same effect on exo-AAV as it does AAV. A recent report described the effect of vitreous aspiration of the mouse eye on AAV8 transduction of retina, showing the feasibility in smaller animal models^41^. Also, exosomes may function to transport attached AAV capsids across the ILM. Heparin-sensitive binding is implicated in the ability of AAV2 to transduce the retina via IVT injection^21^,^42^. It appears that the extent of heparin sulfate proteoglycan binding influences the level of transduction as capsids with lower affinity heparin binding show better transduction^27^. We have recently shown that the uptake of 293T cell-derived EVs into recipient cells can be blocked with heparin^14^,^23^. While no difference in heparin blockade of transduction efficiency was observed *in vitro* between AAV2 and exo-AAV2, slight differences *in vivo* may play a role in our observed enhancement with exo-AAV2. In the future, we may employ strategies such as Pronase treatment of the ILM to compare it effects on enhancement of transduction of retinal layers by AAV and exo-AAV^11^. It may also be useful to track the ILM accumulation of vector by *in situ* hybridization to AAV genomes^21^ from AAV vs. exo-AAV. These possibilities will be the focus of future laboratory investigations.

In summary, exo-AAV is a convenient and robust tool for intravitreal gene transfer to the mouse retina and as such, should facilitate research of several retinal cell types.

## Materials and Methods

### Vector production

We isolated conventional AAV2 and exo-AAV2 vectors from 293T cells, as previously described^14^,^15^. For each production we plated four 15 cm tissue culture dishes with 1.5 × 10^7^ 293T cells each. The next day cells were transfected (on a per plate basis) using the calcium phosphate method, with the adenovirus helper plasmid (pAdΔF6, 26 μg), AAV2 rep/cap plasmid (pH22, 13 μg) and ITR-flanked self-complimentary GFP (10 μg) to induce production of AAV. Plasmids were diluted in a volume of 780 μl of 2.5 mM HEPES containing calcium chloride and then added drop-wise while vortexing into 780 μl 2x HeBS buffer (280 mM NaCl, 50 mM HEPES, 1.5 mM Na_2_HPO_4_, pH 7.04) in a 15 ml Falcon tube. The mixture was incubated at room temperature for 20 min before adding it to cells drop-wise. The day after transfection the medium was changed to DMEM containing 2% FBS. The following day the medium was changed to DMEM containing 2% exosome-free FBS (made by overnight 100,000 g ultracentrifugation to deplete bovine exosomes). Exo-AAV vectors were isolated from the media three days after transfection using differential centrifugation as described before^15^. Briefly, cells were depleted at 300 g for 10 min and 1,500 g for 15 min. Next, larger extracellular vesicles (apoptotic bodies, microvesicles) were depleted by a 20,000 g spin for 60 min. The supernatant of the 20,000 g spin was subjected to 1-h 100,000 g centrifugation using a Type 70 Ti rotor in an Optima L-90K ultracentrifuge (both Beckman Coulter, Indianapolis IN, USA). The exosome pellet was re-suspended in serum-free, antibiotic-free DMEM medium. Conventional AAVs were purified from the cell lysate using iodixanol-gradient ultracentrifugation and buffer exchange to PBS was performed. Exo-AAV and conventional AAV vectors were stored at 4 °C and used the next day for injections. While exo-AAV can be frozen at −80 °C, in the current formulation we noticed some aggregation after freeze/thaw which could impede IVT injections, therefore we stored the preparations short-term at 4 °C until injection. For titration of vectors, we first treated samples with DNase to remove plasmid DNA. Next, we isolated vector nucleic acids from the samples using the Roche High Pure Nucleic Acid viral kit (Roche, Pleasanton, CA, USA), in order to remove PCR inhibitors and nucleases potentially present in exosome preparations and to fully lyse the exosomal membrane. Finally, we quantified AAV vector genomes (vg) in conventional and exo-AAV preparations using TaqMan qPCR with BGH polyA-sequence specific primers and probe^14^.

### Transmission electron microscopy

Cell culture media containing exo-AAVs was collected 4 days after transfection and subjected to serial centrifugations described above. The exosome pellet was fixed with 4% formaldehyde (FA) in PBS for 20 minutes in the ultracentrifuge tube. Next, FA was exchanged to 1X PBS and samples were cryoprotected in 2.3 M sucrose in 1X PBS before it was frozen in liquid nitrogen. Cryosections of the exosome pellet (approximately 80 nm thick) were incubated with 1:10 dilutions of mouse anti-AAV2 antibody, which recognizes intact capsids (American Research Products, Waltham, MA, USA; clone A20), followed by a 5-nm-gold conjugated secondary anti-mouse antibody (Sigma-Aldrich, St. Louis, MO, USA). Images were acquired with a Tecnai G 2 Spirit BioTWIN transmission electron microscope (FEI Company, Hillsboro, OR, USA) at the Harvard Medical School Electron Microscopy Facility. We quantified AAV particle association with exosomes on four 2410 × 1510 nm TEM images, with approximately 50 immunogold particles per image.

### Intravitreal injections

Wild-type C57BL/6J male mice (6–8 weeks old) were purchased from Charles River Laboratories and kept at the Schepens Eye Research Institute (SERI) Animal Facility. All animal procedures were performed in accordance with protocols approved by the institutional animal care and use committees at Schepens Eye Research Institute (Boston, MA) and conformed to the guidelines on the care and use of animals adopted by the Association for Research in Vision and Ophthalmology (Rockville, MD). Animals were anaesthetized with a ketamine/xylazine solution and intravitreal injections were performed with 2 μl of exo-AAV2 or conventional AAV2 vectors at 2 × 10^9^ total vg (both were diluted to a titer of 1 × 10^12^ vg/ml with serum-free, antibiotic-free DMEM medium). Briefly, once animals were anaesthetized, topical 1% tropicamide (Akorn Pharmaceuticals, Illinois, USA) and 0.3% Genteal (Novartis, Texas, USA) was applied to the cornea. Once pupil was dilated, a small incision on the limbus area was made using a 30G needle. Intravitreal injections were then performed using a Nanofil syringe with a 33G blunt needle through the limbal incision, carefully avoiding the lens and directing the tip of the needle to the vitreous cavity around the optic nerve head. After injections animals were treated topically on the cornea with neomycin and polymyxin b sulfates and bacitracin zinc ophthalmic ointment (Bausch and Lomb, FL, USA) and allowed to recover from anesthesia.

### Fundus imaging

Fundus imaging was performed using a Micron III (Phoenix Research Labs, Pleasanton, CA) at 2 and 4 weeks post-injections. Animals were anaesthetized with ketamine/xylazine solution, pupils dilated with 1% tropicamide and topical 0.3% Genteal was applied to the cornea prior to imaging. Images were taken at the maximum light intensity with 5 different gains for each eye to cover variation of GFP intensities. For image analysis we used the same gain settings across all samples.

### Retinal sections and immunofluorescence

Eyes were immediately placed in 4% FA upon enucleation for 30 minutes, washed with PBS and pierced with a 30G needle, then placed back into 4% FA for another 30 minutes. The tissue was washed with PBS before placed in 30% sucrose for 30 minutes at 4 °C. Next, sucrose was washed away and eyes were placed in an embedding capsule (Electron Microscopy Sciences, EMS, No. 70021). The capsule was then filled with O.C.T. media (Tissue Tek, VWR, Radnor, PA), frozen over dry ice and then stored at −80 °C until further use. Frozen sections of 15 μm thickness were cut with a Leica CM 1950 cryostat. Slides were rehydrated using PBS, permeabilized with 10% triton in PBS (wash buffer), then blocked for one hour with normal goat serum (10% serum in wash buffer). Primary antibodies were then incubated for two days in blocking solution. Next, sections were rinsed in wash buffer, treated with the appropriate secondary antibody (Invitrogen) at a concentration of 1:500 diluted in blocking solution for 2 hours. Then, the sections were washed with PBS and incubated with10 μg/mL 4′,6-diamidino-2-phenylindole (DAPI) for 10 minutes, followed by a final PBS wash and mounting with ProLong Gold antifade reagent (Life Technologies, OR, USA). Retinal ganglion cells were stained with antibodies against RNA-binding protein with multiple splicing (RBMPS, 1:100, PhosphoSolutions)^43^, bipolar cells were stained with protein kinase Cα antibody (PKCα, Santa Cruz Biotechnology)^44^ and calcium binding protein 5 antibody (CaBP5, 1:10 courtesy of Prof. Françoise Haeseleer, University of Washington), while amacrine cells were stained by anti-choline acetyltransferase antibody (ChAT, 1:100, Millipore). Although we imaged the slides without the routine use of anti-GFP, in one experiment, we also performed anti-GFP staining (anti-GFP-alexa555, 1:500, Abcam) on some slides (indicated in Fig. 3D legend). Retinal sections were imaged with a Leica TCS SP5 Confocal system and an Olympus FluoView 1000 confocal microscope (Olympus, Center Valley, PA, USA) using a PlanApoN 60x/1.42NA oil-immersion objective.

### Imaging and image analysis

To analyze GFP expression on fundus images, we determined total GFP intensity in the field of view using ImageJ 1.50 g^45^. GFP distribution was analyzed with the Concentric Circles Plugin with the center at the optic disk. To describe GFP expression in retinal sections, we plotted GFP intensity as a function of distance from the inner limiting membrane using the Plot Profile Function. Due to slight differences in tissue thickness between different samples, we binned values into 500 equal bins using MatLab R2010a. GFP intensity was averaged in each bin among different samples and plotted as a function of distance (Fig. 3C,D).

### Quantitative real-time PCR

mRNA was extracted from whole eye cups using the RNeasy kit (Qiagen, Valencia, CA, USA). Real-time PCR was performed in triplicates (on three separate plates) using the TaqMan^®^ gene expression master mix (Thermo Fisher Scientific, USA) on an ABI 7500 Real Time PCR System (Applied Biosystems, Waltham, MA, USA). Relative transcript levels were assessed using the ΔCT method with GAPDH as the reference gene (Taqman Assay Mm99999915_g1; Cat. # 4331182; Life Technologies). Primers used for GFP detection were the following (5′–3′): AGCAAAGACCCCAACCAGAA (forward) and GGCGGCGGTACAGAA (reverse) and probe was from Life Technologies (USA).

### Statistics

To compare means, we first ran a Shapiro Wilk normality test. Where applicable we used two-tailed t-test, for datasets that did not pass normality test, we used two-tailed Mann-Whitney test. p < 0.05 was termed significant.

## Additional Information

**How to cite this article**: Wassmer, S. *et al*. Exosome-associated AAV2 vector mediates robust gene delivery into the murine retina upon intravitreal injection. *Sci. Rep.* **7**, 45329; doi: 10.1038/srep45329 (2017).

**Publisher's note:** Springer Nature remains neutral with regard to jurisdictional claims in published maps and institutional affiliations.

## Supplementary Material

Supplementary Information

## Figures and Tables

**Table 1:** Yield of media-isolated AAV2 vector in vesicle pellets and pellet-depleted media.

Fraction	Titer (vg/ml) Mean +/− SD	Volume	Total yield (titer x volume) Mean +/− SD
20k × g pellet	6.5 × 10^11^ ± 2.55 × 10^11^	0.3 ml	2.0 × 10^11^ ± 7.8 × 10^10^
100k × g pellet	1.4 × 10^12^ ± 1.41 × 10^11^	0.2 ml	2.8 × 10^11^ ± 2.8 × 10^10^
Media after centrifugation steps	8.65 × 10^8^ ± 6.4 × 10^7^	40 ml	3.5 × 10^10^ ± 2.8 × 10^9^

n = 2 independent exo-AAV2 preparations to calculate the mean and standard deviation.

**Table 2:** Comparison of conventional and exosome-associated AAV vectors.

	Conventional AAV2	Exosome-associated AAV2
Production	293T cells, triple plasmid transfection using calcium phosphate method	
Source	Cell lysate	Culture media
Isolation	Iodixanol-gradient ultracentrifugation	Pelleting by ultracentrifugation at 100,000 g
Ease of isolation	Difficult	Easy
Titration	qPCR (vector genomes, vg)	
Total yield/150 mm dish	2 × 10^11^ vg	6 × 10^10^ vg
Titer	3 × 10^12^ vg/mL	1.2 × 10^12^ vg/mL
Composition	Highly purified AAV	AAV, exosomes, some co-pelleting proteins

## References

[b1] Maguire A. M. . Safety and efficacy of gene transfer for Leber’s congenital amaurosis. The New England journal of medicine 358, 2240–2248, doi: 10.1056/NEJMoa0802315 (2008). 18441370 PMC2829748

[b2] Bainbridge J. W. . Effect of gene therapy on visual function in Leber’s congenital amaurosis. The New England journal of medicine 358, 2231–2239, doi: 10.1056/NEJMoa0802268 (2008). 18441371

[b3] Cideciyan A. V. . Human gene therapy for RPE65 isomerase deficiency activates the retinoid cycle of vision but with slow rod kinetics. Proceedings of the National Academy of Sciences of the United States of America 105, 15112–15117, doi: 10.1073/pnas.0807027105 (2008). 18809924 PMC2567501

[b4] Jacobson S. G. . Safety in nonhuman primates of ocular AAV2-RPE65, a candidate treatment for blindness in Leber congenital amaurosis. Human gene therapy 17, 845–858, doi: 10.1089/hum.2006.17.845 (2006). 16942444

[b5] Maguire A. M. . Age-dependent effects of RPE65 gene therapy for Leber’s congenital amaurosis: a phase 1 dose-escalation trial. Lancet 374, 1597–1605, doi: 10.1016/S0140-6736(09)61836-5 (2009). 19854499 PMC4492302

[b6] Park S. W., Kim J. H., Park W. J. & Kim J. H. Limbal Approach-Subretinal Injection of Viral Vectors for Gene Therapy in Mice Retinal Pigment Epithelium. Journal of visualized experiments: JoVE e53030, doi: 10.3791/53030 (2015). 26274541 PMC4544934

[b7] Vacca O. . Using Adeno-associated Virus as a Tool to Study Retinal Barriers in Disease. Journal of visualized experiments: JoVEdoi: 10.3791/52451 (2015). PMC4541578 25938717

[b8] Petrs-Silva H. . High-efficiency transduction of the mouse retina by tyrosine-mutant AAV serotype vectors. Molecular therapy: the journal of the American Society of Gene Therapy 17, 463–471, doi: 10.1038/mt.2008.269 (2009). 19066593 PMC2835095

[b9] Tshilenge K. T. . Vitrectomy Before Intravitreal Injection of AAV2/2 Vector Promotes Efficient Transduction of Retinal Ganglion Cells in Dogs and Nonhuman Primates. Human gene therapy methods 27, 122–134, doi: 10.1089/hgtb.2016.034 (2016). 27229628

[b10] Cehajic-Kapetanovic J., Le Goff M. M., Allen A., Lucas R. J. & Bishop P. N. Glycosidic enzymes enhance retinal transduction following intravitreal delivery of AAV2. Molecular vision 17, 1771–1783 (2011). 21750604 PMC3133842

[b11] Dalkara D. . Inner limiting membrane barriers to AAV-mediated retinal transduction from the vitreous. Molecular therapy: the journal of the American Society of Gene Therapy 17, 2096–2102, doi: 10.1038/mt.2009.181 (2009). 19672248 PMC2814392

[b12] Gyorgy B., Hung M. E., Breakefield X. O. & Leonard J. N. Therapeutic applications of extracellular vesicles: clinical promise and open questions. Annual review of pharmacology and toxicology 55, 439–464, doi: 10.1146/annurev-pharmtox-010814-124630 (2015). PMC4445965 25292428

[b13] Gyorgy B. . Membrane vesicles, current state-of-the-art: emerging role of extracellular vesicles. Cellular and molecular life sciences: CMLS 68, 2667–2688, doi: 10.1007/s00018-011-0689-3 (2011). 21560073 PMC3142546

[b14] Maguire C. A. . Microvesicle-associated AAV vector as a novel gene delivery system. Molecular therapy: the journal of the American Society of Gene Therapy 20, 960–971, doi: 10.1038/mt.2011.303 (2012). 22314290 PMC3345986

[b15] Gyorgy B., Fitzpatrick Z., Crommentuijn M. H., Mu D. & Maguire C. A. Naturally enveloped AAV vectors for shielding neutralizing antibodies and robust gene delivery *in vivo*. Biomaterials 35, 7598–7609, doi: 10.1016/j.biomaterials.2014.05.032 (2014). 24917028 PMC4104587

[b16] Hudry E. . Exosome-associated AAV vector as a robust and convenient neuroscience tool. Gene therapy 23, 380–392, doi: 10.1038/gt.2016.11 (2016). 26836117 PMC4824662

[b17] El Andaloussi S., Lakhal S., Mager I. & Wood M. J. Exosomes for targeted siRNA delivery across biological barriers. Advanced drug delivery reviews 65, 391–397, doi: 10.1016/j.addr.2012.08.008 (2013). 22921840

[b18] Yang T. . Exosome delivered anticancer drugs across the blood-brain barrier for brain cancer therapy in Danio rerio. Pharmaceutical research 32, 2003–2014, doi: 10.1007/s11095-014-1593-y (2015). 25609010 PMC4520542

[b19] De Silva S. R. . Single residue AAV capsid mutation improves transduction of photoreceptors in the Abca4−/− mouse and bipolar cells in the rd1 mouse and human retina *ex vivo*. Gene therapy, doi: 10.1038/gt.2016.54 (2016). PMC5097463 27416076

[b20] Pillay S. . An essential receptor for adeno-associated virus infection. Nature 530, 108–112, doi: 10.1038/nature16465 (2016). 26814968 PMC4962915

[b21] Woodard K. T., Liang K. J., Bennett W. C. & Samulski R. J. Heparan Sulfate Binding Promotes Accumulation of Intravitreally-Delivered Adeno-Associated Viral Vectors at the Retina for Enhanced Transduction but Weakly Influences Tropism. Journal of virology, doi: 10.1128/JVI.01568-16 (2016). PMC5068537 27558418

[b22] Balaj L. . Heparin affinity purification of extracellular vesicles. Scientific reports 5, 10266, doi: 10.1038/srep10266 (2015). 25988257 PMC4437317

[b23] Atai N. A. . Heparin blocks transfer of extracellular vesicles between donor and recipient cells. Journal of neuro-oncology 115, 343–351, doi: 10.1007/s11060-013-1235-y (2013). 24002181 PMC3856724

[b24] Christianson H. C., Svensson K. J., van Kuppevelt T. H., Li J. P. & Belting M. Cancer cell exosomes depend on cell-surface heparan sulfate proteoglycans for their internalization and functional activity. Proceedings of the National Academy of Sciences of the United States of America 110, 17380–17385, doi: 10.1073/pnas.1304266110 (2013). 24101524 PMC3808637

[b25] Vacca O. . AAV-mediated gene delivery in Dp71-null mouse model with compromised barriers. Glia 62, 468–476, doi: 10.1002/glia.22617 (2014). 24382652

[b26] Spiteri Cornish K. . Vitrectomy with internal limiting membrane peeling versus no peeling for idiopathic full-thickness macular hole. Ophthalmology 121, 649–655, doi: 10.1016/j.ophtha.2013.10.020 (2014). 24314837

[b27] Dalkara D. . *In vivo*-directed evolution of a new adeno-associated virus for therapeutic outer retinal gene delivery from the vitreous. Science translational medicine 5, 189ra176, doi: 10.1126/scitranslmed.3005708 (2013). 23761039

[b28] Ramachandran P. S. . Evaluation of Dose and Safety of AAV7m8 and AAV8BP2 in the Non-Human Primate Retina. Human gene therapy, doi: 10.1089/hum.2016.111 (2016). PMC5312498 27750461

[b29] Petrs-Silva H. . Novel properties of tyrosine-mutant AAV2 vectors in the mouse retina. Molecular therapy: the journal of the American Society of Gene Therapy 19, 293–301, doi: 10.1038/mt.2010.234 (2011). 21045809 PMC3034844

[b30] Mowat F. M. . Tyrosine capsid-mutant AAV vectors for gene delivery to the canine retina from a subretinal or intravitreal approach. Gene therapy 21, 96–105, doi: 10.1038/gt.2013.64 (2014). 24225638 PMC3880610

[b31] Kay C. N. . Targeting photoreceptors via intravitreal delivery using novel, capsid-mutated AAV vectors. PloS one 8, e62097, doi: 10.1371/journal.pone.0062097 (2013). 23637972 PMC3637363

[b32] Vandenberghe L. H. & Auricchio A. Novel adeno-associated viral vectors for retinal gene therapy. Gene therapy 19, 162–168, doi: 10.1038/gt.2011.151 (2012). 21993172

[b33] Boyd R. F. . Photoreceptor-targeted gene delivery using intravitreally administered AAV vectors in dogs. Gene therapy 23, 400, doi: 10.1038/gt.2016.10 (2016). 27052928

[b34] Giove T. J., Sena-Esteves M. & Eldred W. D. Transduction of the inner mouse retina using AAVrh8 and AAVrh10 via intravitreal injection. Experimental eye research 91, 652–659, doi: 10.1016/j.exer.2010.08.011 (2010). 20723541 PMC2962726

[b35] Wiley R. D. & Gummuluru S. Immature dendritic cell-derived exosomes can mediate HIV-1 trans infection. Proceedings of the National Academy of Sciences of the United States of America 103, 738–743, doi: 10.1073/pnas.0507995103 (2006). 16407131 PMC1334656

[b36] Feng Z. . A pathogenic picornavirus acquires an envelope by hijacking cellular membranes. Nature 496, 367–371, doi: 10.1038/nature12029 (2013). 23542590 PMC3631468

[b37] Yang Y. . Exosomes mediate hepatitis B virus (HBV) transmission and NK-cell dysfunction. Cellular & molecular immunology, doi: 10.1038/cmi.2016.24 (2016). PMC5423088 27238466

[b38] Ran L. . Delivery of oncolytic adenovirus into the nucleus of tumorigenic cells by tumor microparticles for virotherapy. Biomaterials 89, 56–66, doi: 10.1016/j.biomaterials.2016.02.025 (2016). 26950165

[b39] Dudus L. . Persistent transgene product in retina, optic nerve and brain after intraocular injection of rAAV. Vision research 39, 2545–2553 (1999). 10396623 10.1016/s0042-6989(98)00308-3

[b40] Mulcahy L. A., Pink R. C. & Carter D. R. Routes and mechanisms of extracellular vesicle uptake. Journal of extracellular vesicles 3, doi: 10.3402/jev.v3.24641 (2014). PMC4122821 25143819

[b41] Da Costa R. . A Novel Method Combining Vitreous Aspiration and Intravitreal AAV2/8 Injection Results in Retina-Wide Transduction in Adult Mice. Investigative ophthalmology & visual science 57, 5326–5334, doi: 10.1167/iovs.16-19701 (2016). 27784063

[b42] Boye S. L. . Impact of Heparan Sulfate Binding on Transduction of Retina by Recombinant Adeno-Associated Virus Vectors. Journal of virology 90, 4215–4231, doi: 10.1128/JVI.00200-16 (2016). 26865709 PMC4810560

[b43] Kwong J. M., Caprioli J. & Piri N. RNA binding protein with multiple splicing: a new marker for retinal ganglion cells. Investigative ophthalmology & visual science 51, 1052–1058, doi: 10.1167/iovs.09-4098 (2010). 19737887 PMC3979483

[b44] Wassle H., Yamashita M., Greferath U., Grunert U. & Muller F. The rod bipolar cell of the mammalian retina. Visual neuroscience 7, 99–112 (1991). 1718403 10.1017/s095252380001097x

[b45] Schneider C. A., Rasband W. S. & Eliceiri K. W. NIH Image to ImageJ: 25 years of image analysis. Nature methods 9, 671–675 (2012). 22930834 10.1038/nmeth.2089 PMC5554542

