# Supplementary material for: Exosome-associated AAV2 vector mediates robust gene delivery into the murine retina upon intravitreal injection
Source: Sci Rep. 2017 Mar 31;7:45329. doi: 10.1038/srep45329 (PMC5374486; doi:10.1038/srep45329)

Supplementary Information

Exosome-associated AAV2 vector mediates robust gene delivery into the murine retina upon intravitreal injection

Sarah Wassmer[1], Livia Carvalho[1], Bence György[2,3#], Luk H. Vandenberghe[1#], Casey A. Maguire[3#]

Affiliations:

[1] Harvard Stem Cell Institute, Harvard University, Cambridge, MA, USA, Grousbeck Gene Therapy Center, Schepens Eye Research Institute and Massachusetts Eye and Ear, Boston, MA, USA; Ocular Genomics Institute, Department of Ophthalmology, Harvard Medical School, Boston, MA, USA

[2] Department of Neurobiology and Howard Hughes Medical Institute, Harvard Medical School, 220 Longwood Avenue, Boston, 02115 MA, USA

[3] Department of Neurology, Massachusetts General Hospital and NeuroDiscovery Center, Harvard Medical School, Building 149, Charlestown, Boston, 02129 MA, USA

[#]Co-corresponding authors:

Casey A. Maguire, PhD: cmaguire@mgh.harvard.edu

Luk H. Vandenberghe, PhD: Luk_Vandenberghe@meei.harvard.edu

Bence György, MD, PhD: Bence_Gyorgy@hms.harvard.edu

Supplementary Materials and Methods

Luciferase assays

HeLa transductions. Exo-AAV2 and conventional AAV2 encoding firefly luciferase was added to HeLa cells for 2 hours at the indicated vg/cell. Cells were then incubated for 48 h before performing a luciferase assay using Bright-Glo™ Luciferase reagent (Promega, Madison, WI). For heparin inhibition experiments, vectors were incubated with heparin (1-200 µg/mL) for 30 minutes at 37 °C before adding to cells. Luciferase values for each sample, expressed in relative light units (RLUs) were plotted as a percentage of the AAV transduction sample without heparin.

AAVR experiment. AAVR wildtype, knock out and over-expressing cells lines (HEK 293 cells) were kindly provided by the Carette laboratory at Stanford, and described here (see reference 20 of main text, Pillay *et al.* 2016). All three lines (passage #11) were plated at 25,000 cells per well in a 96-well format (on poly-l-lysine coated wells). The following day, AAV2-CBA-FLuc and Exo-AAV2-CBA-FLuc at an MOI of 1,000 was added to each well, diluted in serum free media (DMEM with 4.5g/L glucose, l-glutamine, sodium pyruvate and penicillin/streptomycin, Corning). After two hours, the media was changed to complete media containing 10%FBS (Thermo Scientific). 48 hours post application of virus, the media was changed again to complete media, and then 24 hours later, the media was removed and 20ul of lysis buffer (Promega) was added to each well. The plate was then frozen at -80C for 30 minutes and transferred to 37C for 15 minutes. Luminescence was read using the SynergyH1 BioTek plate reader after 100ul of luciferase substrate buffer (Tris-HCl, $MgCl_2$, ATP [Life Technologies] and D-luciferin [Caliper Life Sciences]) was added to each well.

Supplementary Figure S1. Additional Images of exo-AAV2 injected mice. Panel A-C show the ciliary body of an exo-AAV2 injected animal. Within the section, both the superior and inferior part of the ciliary body expressed GFP. Panel D-F shows PKC-alpha staining (red) of an exo-AAV2 injected animal. It is evident that PKC-alpha positive bipolar cells are transduced by exo-AAV2 and express GFP (arrow), the photoreceptor nuclei are also transduced by exo-AAV2 (arrow head). Scale bar= 20 µm.

Supplementary Figure S2. Detection of photoreceptor transduction of intravitreally injected exo-AAV2 in 4 out of 5 mice. White arrows point to photoreceptor nuclei, while white arrowheads point to GFP filled inner segments of these cells. Each panel depicts an individual mouse retina. The lower right panel depicts the mouse which appeared to have received a suboptimal injection and did not demonstrate any photoreceptor GFP expression. Scale bar= 100 µm.

Supplementary Figure S3. Transduction of Müller cells by exo-AAV2. Direct immunofluorescence of GFP in the AAV2 injected animal with highest transgene expression and a exo-AAV2 injected animal shows GFP expression in a peripheral section through the retina. The exo-AAV2 animal has co-localization with glutamine synthetase (GS6) expressing Müller glia at the cell end-feet (arrow). Scale bar = 50µm.

Supplementary Figure S4. Transduction by exo-AAV2, similar to AAV2, is dependent on the universal AAV receptor (AAVR). Wildtype AAVR expressing cells, AAVR knock-out and AAVR over-expressing cell lines (AAVR Rescue) were transduced by AAV2 or exo-AAV2 encoding firefly luciferase at 1,000 vg/cell and subsequently luciferase expression measured with a luminometer. For each vector, we calculated the percentage transduction on wild type cells.

Supplementary Figure S5. Transduction of HeLa cells with AAV2 and exo-AAV2. (A) HeLa cells were transduced with vectors encoding for firefly luciferase at 1,000 and 10,000 vector genomes of AAV per cell (vg/cell) for 2 hours. Luciferase activity was measured 48 hours later from cell lysates. (B) Vectors were preincubated with different concentrations of heparin for 30 minutes at 37 °C before adding them to HeLa cells. Luciferase activity was measured 48 hours later from cell lysates. *** $p<0.001$, two tailed t-test.

Supplementary Fig. S1

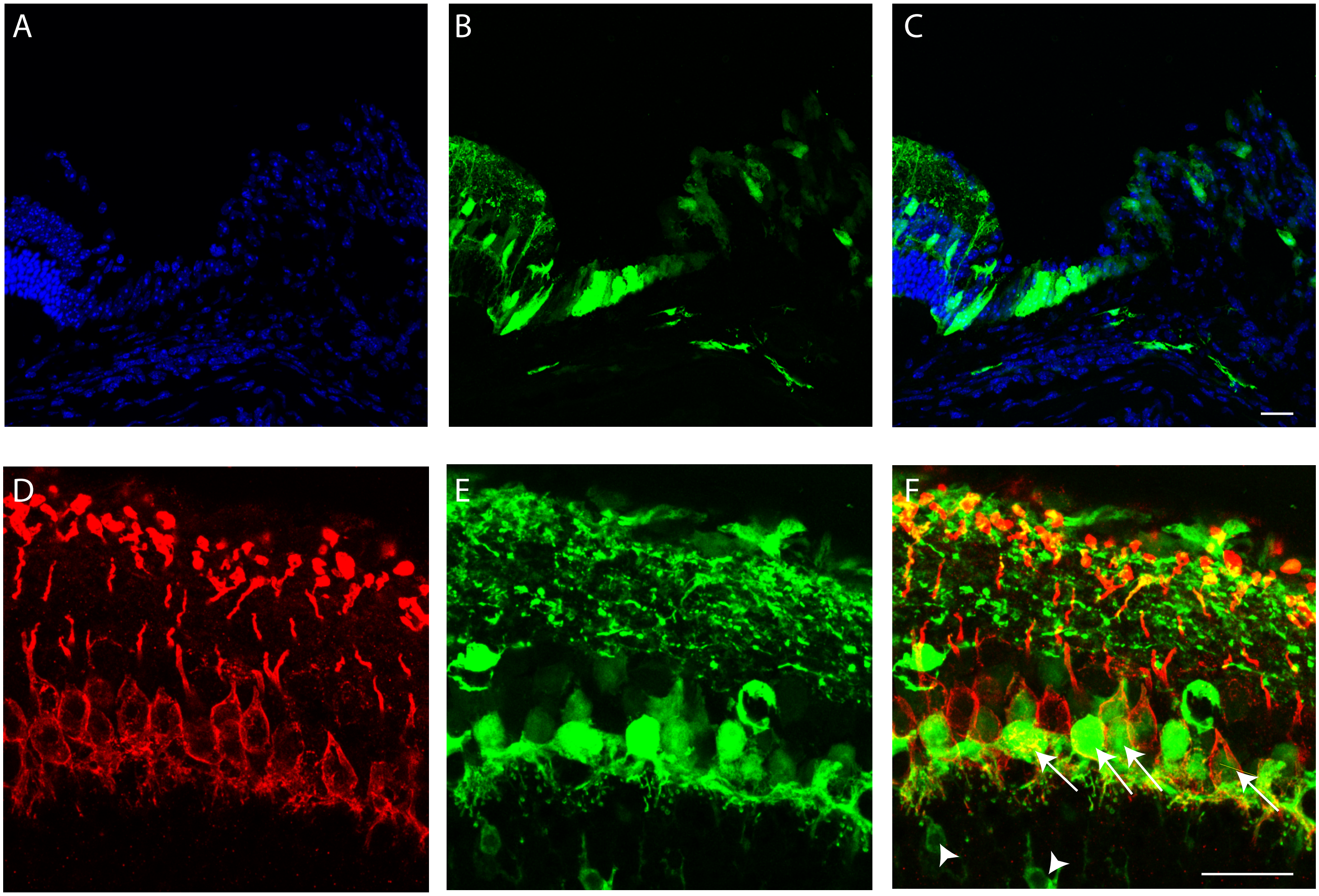

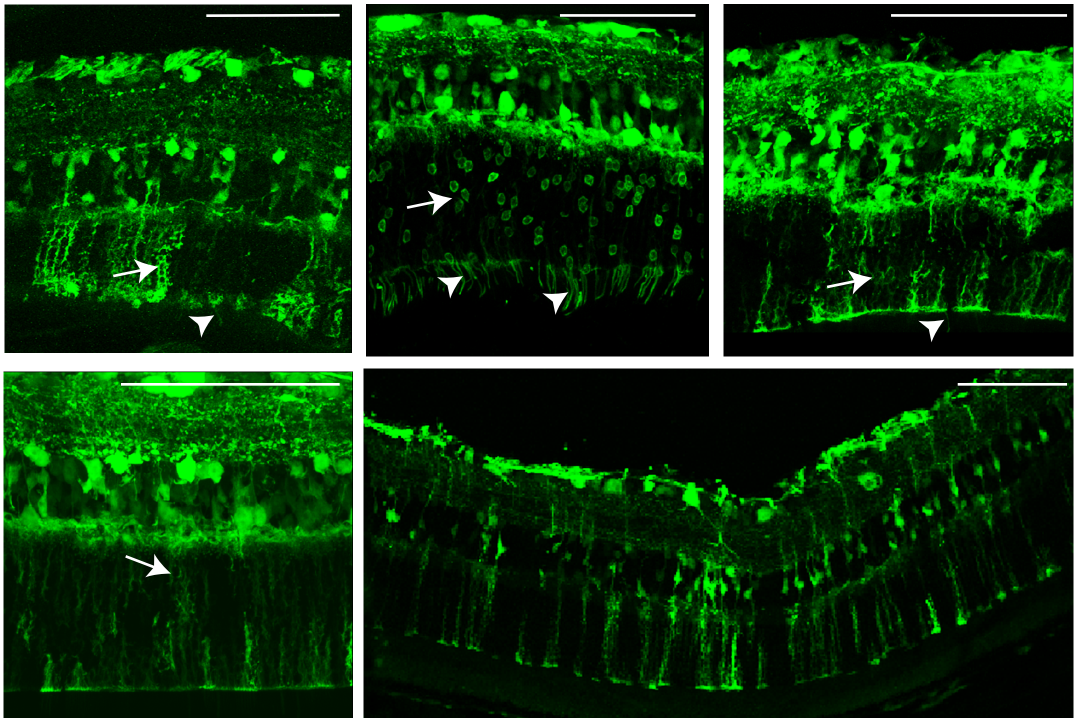

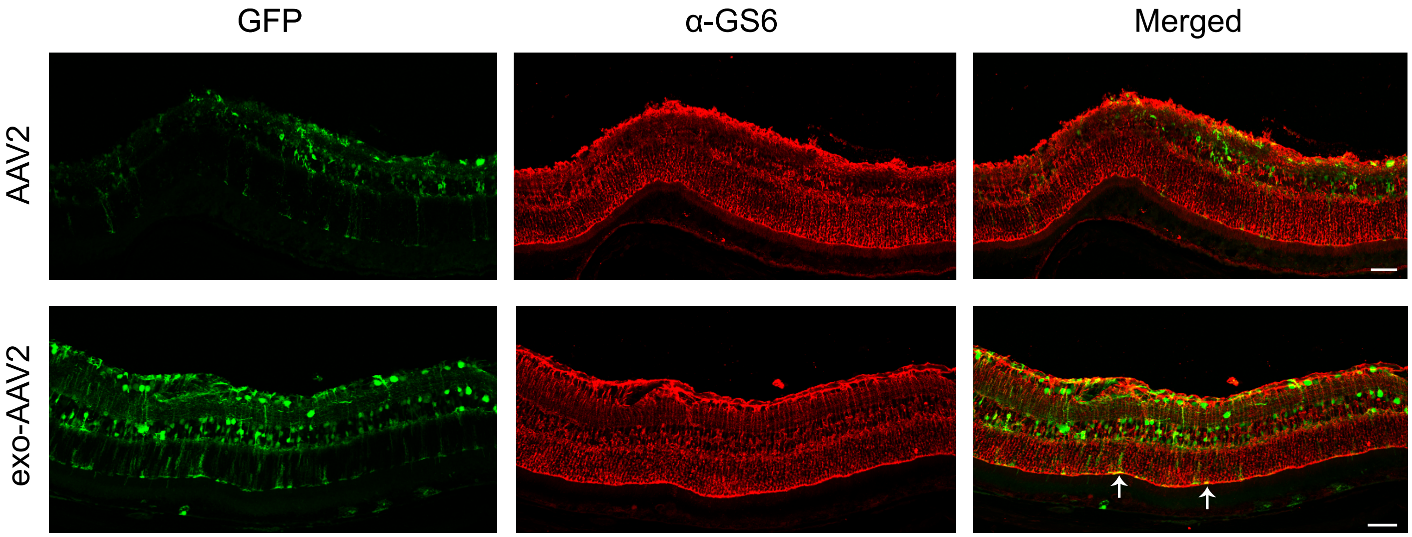

Supplementary Fig. S3

Supplementary Fig S4

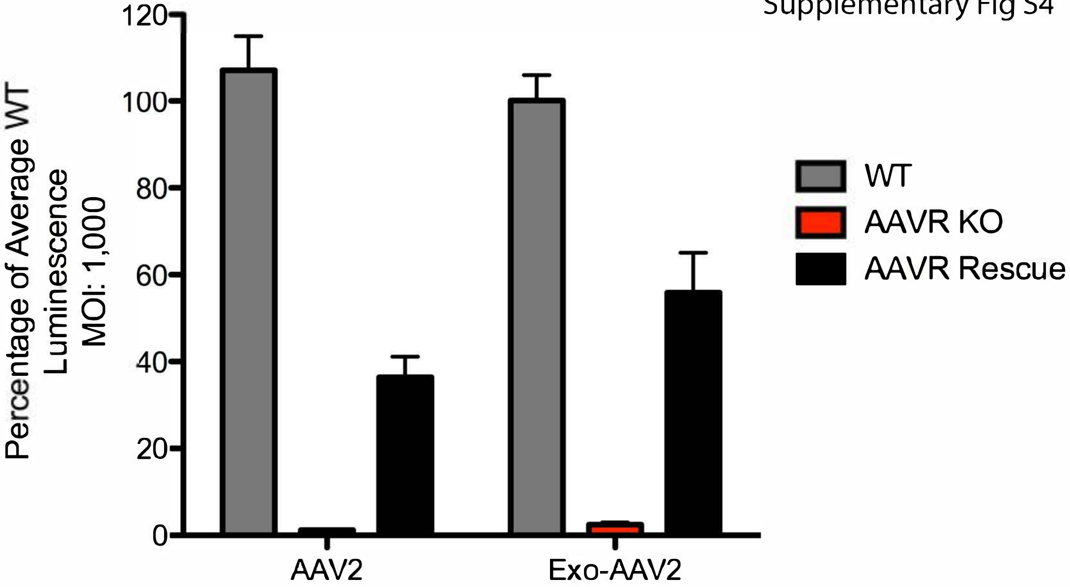

Supplementary Fig. S5

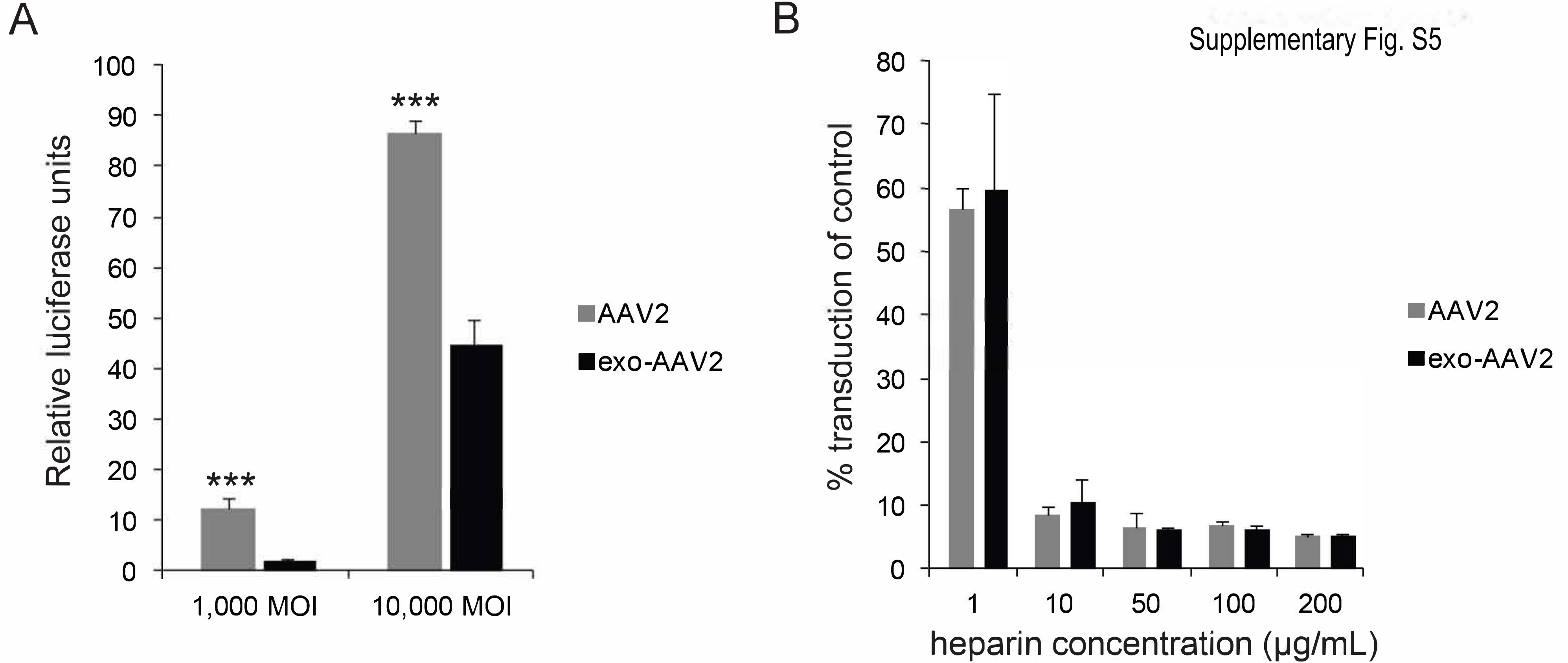

Supplement: Supplementary Information [file srep45329-s1.pdf]
